# The Extracellular Protein, Transthyretin Is an Oxidative Stress Biomarker

**DOI:** 10.3389/fphys.2019.00005

**Published:** 2019-01-24

**Authors:** Meesha Sharma, Sheeza Khan, Safikur Rahman, Laishram Rajendrakumar Singh

**Affiliations:** ^1^Dr. B.R. Ambedkar Center for Biomedical Research, University of Delhi, New Delhi, India; ^2^Department of Medical Biotechnology, Yeungnam University, Gyeongsan, South Korea

**Keywords:** transthyretin, oxidative stress, cryptic protease activity, thyroxin, retinol binding protein, biomarker

## Abstract

The extracellular protein, transthyretin is responsible for the transport of thyroxin and retinol binding protein complex to the various parts of the body. In addition to this transport function, transthyretin has also been involved in cardiovascular malfunctions, polyneuropathy, psychological disorders, obesity and diabetes, etc. Recent developments have evidenced that transthyretin has been associated with many other biological functions that are directly or indirectly associated with the oxidative stress, the common hallmark for many human diseases. In this review, we have attempted to address that transthyretin is associated with oxidative stress and could be an important biomarker. Potential future perspectives have also been discussed.

## Introduction

Transthyretin (TTR), also known as prealbumin is a 55 kDa homo-tetrameric protein found in plasma and cerebrospinal fluid. It consists of four identical subunits, with each subunit consisting of 127 amino acid residues (Gonzalez and Offord, 1971; Kanda et al., 1974). TTR structure is rich in β-sheets with four binding sites; two for thyroxine and two for retinol-RBP complex (Ingbar, 1958; Naylor and Newcomer, 1999). It is encoded by a 7 kb gene (comprising of four exon and three introns) located at chromosome 18q11.2– q12.1 (Tsuzuki et al., 1985; Sparkes et al., 1987). It is primarily responsible for the transport of thyroxin and retinol-retinol binding complex (RBP-complex) to different parts of the body and brain (Raz and Goodman, 1969; Power et al., 2000). The major site of serum TTR synthesis is liver with normal concentration in the range of 0.2–0.4 mg/ml and half-life of 2 days. In central nervous system, TTR is expressed in choroid plexus and is released into the cerebrospinal fluid with concentration in the range of 0.02–0.04 mg/ml (Soprano et al., 1985). In addition to plasma and cerebrospinal fluid, it is also expressed in the endothelial cells of Islets of Langerhans, retinal and ciliary pigment epithelia in trace amounts (Cavallaro et al., 1990; Kawaji et al., 2005; Westermark and Westermark, 2008). TTR may also undergo oligomerization and such TTR oligomers are specifically picked up by cardiomyocytes, neuronal and kidney cells leading to organ malfunctions (Colon and Kelly, 1992). Deficiency of the normal function of TTR has been known to be associated with obesity and diabetes (Yang et al., 2005). The roles of TTR in the central nervous system, especially in cognition and memory, psychological health and emotion have also been widely understood (Fleming et al., 2007; Brouillette and Quirion, 2008). The oligomeric form of the TTR has been found to be involved in the pathophysiology of various diseases including atherosclerosis, familial amyloidosis polyneuropathy (Costa et al., 1978), senile systemic amyloidosis (Westermark et al., 1990), familial amyloidosis cardiomyopathy (Jacobson et al., 1997; Yokoyama et al., 2015; Sant’anna et al., 2017) etc. Although main function of transthyretin is the transport of thyroxine and retinol bound to retinol binding protein (RBP), there are many other biological roles of TTR that are directly or indirectly related to anti-oxidant and oxidant properties and could be an important oxidative stress biomarker or therapeutic target. For instance, (i) TTR level correlates well with reactive oxygen species (ROS) or reactive nitrogen species (RNS) (Saito et al., 2005; Fong and Vieira, 2013); (ii) TTR gene expression is regulated by stress hormone, glucocorticoid and sex hormone, estradiol (Li et al., 2011; Martinho et al., 2012); (iii) Even though TTR is an extracellular protein, it can induce oxidative stress in endoplasmic stress (ER) and hence involved in unfolded protein response (UPR) (Teixeira et al., 2006; Genereux et al., 2015; Chen et al., 2016); (iv) The oligomeric forms of TTR also plays an important role in inducing oxidative stress and could be involved in different pathophysiologies (Hammarström et al., 2002; Zhao et al., 2013). In the light of these observations, this review article has been designed to discuss that TTR is associated with oxidative stress and has implications for potential disease specific biomarker.

## TTR is a Neuronal Stress Biomarker

It has already been understood that oxidative stress is one primary cause of Alzheimer’s disease (AD) and many other neurodegenerative diseases (Marques et al., 2003). Recent advances have unveiled that such a cause of oxidative stress has a good correlation with the role of TTR. This is evident from various studies. First, TTR level is upregulated in patients with neurodegenerative disorders (Li et al., 2011) wherein oxidative stress is the common cause of the pathophysiology. Because quantitative real time PCR of TTR mRNA and western blot analysis, have shown that primary neurons from AD mice exhibit upregulation of TTR level as compared to non-demented age-matched individuals or control mice (Li et al., 2011). Second, TTR expression is directly regulated by sex hormones (e.g., estradiol) or stress hormones (e.g., glucocorticoids) in neuronal cells (Martinho et al., 2012). For instance, when rat choroid plexus and choroid plexus epithelial cell lines were incubated with varying concentration of hydrocortisone and estradiol (E2) (0, 10, 100, or 1,000 nM) for 6, 12, 18, 24 and 36 h (Martinho et al., 2012), there was increase in TTR protein and TTR mRNA levels in a concentration dependent manner of the hormones. Similarly, incubation of the cells with respective receptor antagonists results in the suppression of TTR induction. In another experiment, they also analyzed the level of corticosterone in liver, choroid plexus and cerebrospinal fluid of adult rats in response to chronic and acute stress. Stress was induced by increasing the animal density. It was observed that the given treatments drive the upregulation of expression of TTR. In another development, based on *in silico* study, Wakasugi et al. (1986) demonstrated that rat TTR gene contains a glucocorticoid-responsive element in its 3′ region of the first intron (Wakasugi et al., 1986) and this element is conserved in humans as well (Sasaki et al., 1985). Thus, it was concluded that upregulation of TTR expression by glucocorticoid treatments is *via* glucocorticoid-responsive element. Taken together, the results indicate that TTR has a close association with the level of oxidative stress and hence might consequently contribute to the pathogenicity of neurodegeneration.

Third, other studies also reported that TTR has the ability to suppress or remove β-amyloid deposits from neuronal tissues (Buxbaum et al., 2008) making TTR a crucial target for the therapeutic intervention of AD. In fact, direct evidence of the involvement of TTR in AD stems from the identification of physical interaction between TTR and Aβ (Gimeno et al., 2017). Mechanistically, TTR present in the cerebrospinal fluid could sequesters β-amyloid and inhibits the oligomerization and plaque formation (Schwarzman et al., 1994). It is believed that TTR uses its cryptic protease activity to proteolyze Aβ into smaller non-amyloidogenic fragments (Costa et al., 2008; Silva et al., 2017). In another development, recent study further revealed that TTR has higher affinity to Aβ aggregates rather than the fibrils and bind to these pre-toxic aggregates in a chaperon-like manner in both the extracellular and intracellular environment (Buxbaum et al., 2008). It has also been understood the higher the binding affinity between TTR and Aβ, the higher is the inhibitory potential because stabilizers that increase TTR tetramer stability augments the inhibitory effect (Costa et al., 2008; Ribeiro et al., 2012). Similarly, few TTR mutants that is more stable than the Wt TTR has been shown to exhibit more disaggregating potential than Wt TTR (Costa et al., 2008).

It has been known that major cytotoxicity of deposition of β-amyloid is oxidative stress (Butterfield et al., 2001). Since there exists a good correlation between oxidative stress and TTR expression, we speculate that oxidative stress induces glucocorticoids which in turn increase TTR expression via its action on the glucocorticoid receptors. The increased level of TTR will further help to deal with the β-amyloid deposits bringing about its role in preventing AD (Nilsson et al., 2018). In addition to AD, there are a large number of neuronal disorders due to oxidative stresses. These include psychological (e.g., depression), movement disorder (e.g., Parkinson), cognitive disorders etc. Therefore, possibility of the association between these diseases and TTR level may be exploited as a potential biomarker (or therapeutic target) for such disorders.

## Cryptic Protease Activity of Transthyretin Induces Oxidative Stress by Cleaving Apo A-1

High-density lipoprotein (HDL) complex is responsible for reverse cholesterol efflux and cholesterol transport from cells and tissues back to liver (Gordon et al., 1989). Besides cholesterol efflux, HDL also exhibit anti-oxidant activity by forming complex with many anti-oxidant enzymes like paraoxonase, platelet-activating factor acetylhydrolase, glutathione peroxidase, lipid transfer proteins like lecithin: cholesterol acyl transferase, cholesterol ester transfer protein, Apolipoprotein A-I (ApoA-I) and 1-palmitoyl-2-oleoyl-phosphatidylcholine. Among these anti-oxidant enzymes, Apo A-I is the major anti-oxidant and anti-inflammatory component associated with HDL (Navab et al., 2000). It employs anti-oxidant activity by eliminating lipid hydroperoxides from low-density lipoproteins (LDL) and anti-inflammatory properties by shutting down the expression of adhesion molecules (Navab et al., 2000).

**FIGURE 1:**
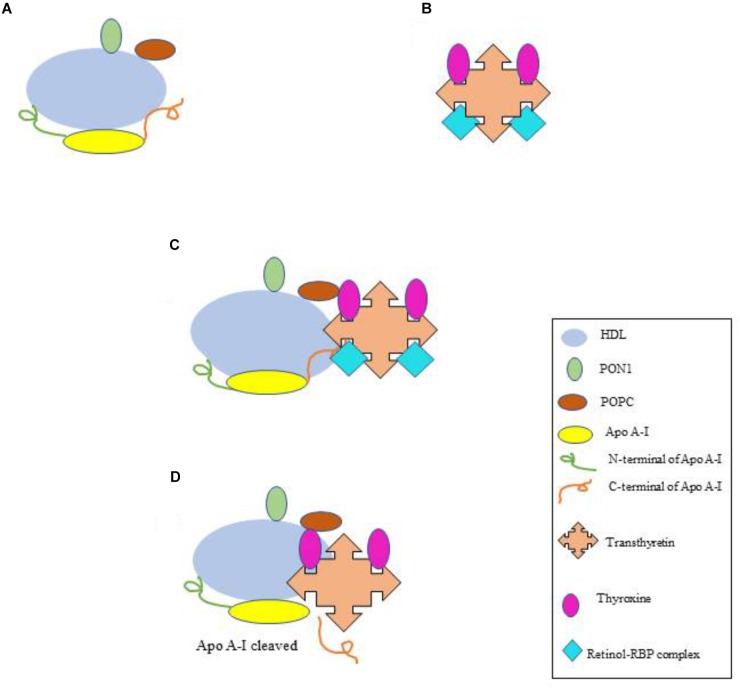
Cryptic protease activity of TTR and cleavage of Apo A-I. **(A)** HDL complex associated with the antioxidant enzymes. **(B)** TTR protein bound to thyroxine and retinol-RBP complex. **(C)** HDL complex with TTR. TTR exhibits no cryptic protease activity until it is bound to retinol-RBP complex. **(D)** Cryptic protease activity of TTR when retinol-RBP complex is not bound, Apo A-I is cleaved into N and C-terminal. HDL, high density lipoprotein; PON1, paraoxonase; POPC, 1-palmitoyl-2-oleoyl-phosphatidylcholine; RBP, retinol binding protein; TTR, transthyretin.

One important protein that affects the anti-oxidant property of HDL is the serum protein, TTR 1-2% of serum TTR is associated with HDL molecules (Sousa et al., 2000). As mentioned above, TTR transports thyroxine and retinol bound to RBP. In the absence of retinol-RBP complex, TTR occasionally exhibit its cryptic protease function (Liz et al., 2004). This activity of TTR brings about specific cleavage of Apo A-I resulting in the loss of anti-oxidant function of HDL (Liz et al., 2007; Podrez, 2010). Figure 1 illustrates the mechanism of how TTR acts to cleave the Apo A-1. Immunologically, the proteolyzed product of Apo A-I acts as a pro-inflammatory molecule that further adds in oxidative stress (Navab et al., 2000). In another development, both the proteolyzed product of apo A-I i.e. N-terminal and C-terminal domains are observed to be amyloidogenic (de Sousa et al., 2000). Since, amyloids or proteins aggregates are one important basic cause of oxidative stress (Abramov et al., 2004), the formation of the amyloidogenic species will further augment the magnitude of the oxidative stress.

Interestingly, Sousa and his group (2004) recently reported that the cryptic protease activity of TTR was seen only when RBP-retinol complex was not bound to TTR (Liz et al., 2004) (Figure 1). This indicates that such type of oxidative stress (due to cryptic protease activity of TTR) may perhaps be related to the retinol deficiency and hence diseases associated with it (Basu et al., 1989). Therefore, the protease activity of TTR can be a potential biomarker for oxidative stress as well as for diseases associated with retinol deficiency. Furthermore Apo-A I deficiency (due to the cleavage by TTR) make HDL unable to remove cholesterol from the tissues. This will eventually result in the atherosclerotic plaque formation. Thus TTR cryptic protease activity and associated oxidative stress may further by employed as a biomarker for cardiovascular disorders.

## TTR is Glutathionylated

Glutathiol (GSH) is a low molecular weight thiol group present in all the cells and serum. GSH also acts as a good indicator of cellular redox state and anti-oxidant defence (Dalle-Donne et al., 2009). The antioxidant property of GSH is mediated by glutathione peroxidase as it oxidizes GSH to GSSG thereby reducing hydrogen peroxide and lipid hydroperoxides (Toborek and Hennig, 1994). Protein-glutathionylation is a redox-mediated post-translational modification, which involves conjugation of a glutathione with a cysteine thiol group on the proteins (Ghezzi, 2005). Glutathionylation not only plays a critical role in many important biological functions (regulation of metabolic pathways, calcium homeostasis, signal transduction, cytoskeleton remodeling, inflammation and protein folding) but also is involved in oxidative stress (Kaplowitz, 1981). The involvement in oxidative stress may stem from at least in two viewpoints. Large scale adduct formation with cysteine group in proteins will eventually lead to deficiency of GSH levels making the system difficult to handle the oxidative stress. Alternatively, once GSH has been bound to cysteine residues in proteins, there is release of free electron (Ghezzi, 2005) that consequently help to generate free radicals thereby inducing oxidative stress. Interestingly, TTR has been reported to be glutathionylated under certain conditions. This was revealed from a study conducted by Ando and his group (1998) to determine the *in vivo* behavior of transthyretin in blood using electrospray ionization mass spectrometry analysis coupled with high-pressure liquid chromatography (HPLC) (Terazaki et al., 1998). Purified TTR from normal subjects was injected into the rats and after 3 h, blood and the urine were analyzed by measuring free or modified TTR. Lower level of free TTR in blood and no TTR secretion into the urine were observed (Terazaki et al., 1998) indicating that major fraction of TTR have been modified by glutathione. Inside the cell, glutathionylation is not restricted to TTR alone but also occurs to many other proteins, the results indicate that TTR indeed contributes to the oxidative stress generated due to protein glutathionylation.

Escher et al. (2007) reported that the levels of glutathionylated form of TTR are inversely correlated in patients with Mycosis fungoides (MF) or non-Hodgkin’s lymphoma (Escher et al., 2007). It is worth noting that MF is associated with the genetic polymorphism in genes involved in the regulation of oxidative stress (Lightfoot et al., 2006). The results hint that the development of MF is because of oxidative stress originated through genetic or post-translational modifications. Therefore, the glutathionylated forms of TTR may be a potential biomarker for early diagnosis or therapeutic target for MF.

## TTR Oligomers as Multiple Biomarkers

**FIGURE 2:**
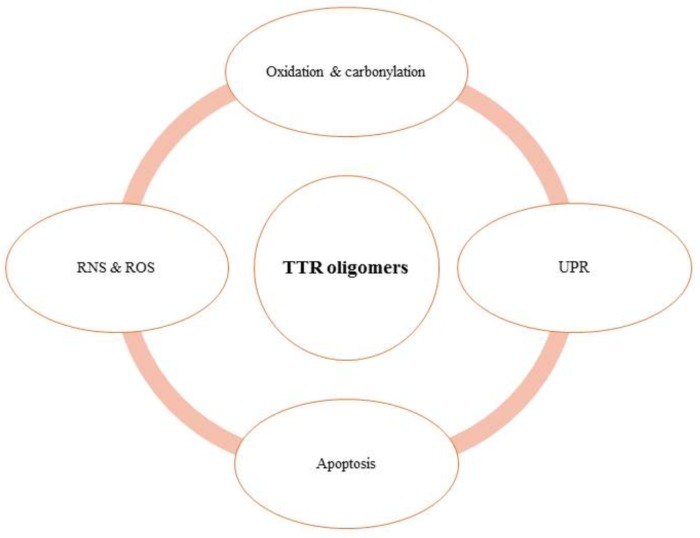
TTR oligomers are linked with various pathophysiological consequences.

It has been well understood that dissociation of TTR oligomer is the rate-limiting step to TTR amyloidosis because dissociation results in the exposition of important sites for oligomerization (Colon and Kelly, 1992; Sousa et al., 2001). Similarly, mutations in TTR disrupt its tetramer and thus form toxic oligomers (Hammarström et al., 2002). The toxic TTR oligomers are believed to preferentially deposit in the extracellular matrix (ECM) of hepatocytes or neuronal cells leading to the development of familial amyloidosis, which encompass FAP (Familial Amyloid Polyneuropathy) (Benson and Kincaid, 2007; Saraiva et al., 2012) and FAC (Familial Amyloid Cardiomyopathy) (Costa et al., 1978). Mechanistically, large deposition of such TTR oligomers in the cardiac and neuronal cells results in the tissue injury that ultimately lead to the increase in inflammatory response (Ton et al., 2014). Since oligomers are known to induce oxidative stress in cells and inflammatory response is going to add more impact on oxidative stress, accumulation of the oligomers eventually results in organ failure or tissue damage due to massive oxidative stress. Although in general oligomers are the exact cause or consequences of such oxidative stress is not clearly understood, it is certainly possible that a positive feedback loop is formed wherein oxidation causes more oligomerization of TTR, which in turn causes more TTR oxidation. In addition to cardiomyopathy and polyneuropathy, this feedback loop so formed (in case of TTR oligomers), may affect following consequences as outlined in Figure 2 leading to involvement of TTR in different pathophysiologies or various biological processes. Following sections will describe the involvement of TTR oligomers in each of the consequences.

### TTR Oligomer Is Related to Reactive Nitrogen Species

In addition to the ROS like superoxide radicals, hydrogen peroxide etc., (RNS) including nitrate and nitrite ions also plays a major role in oxidative stress. Fong and Vieira (2013) gave the first evidence for the increased production of RNS in presence of TTR aggregates in two different human cell lines, epidermoid (A431) and schwannoma (sNF94.3) (Fong and Vieira, 2013). Moreover, the authors also observed that the cells treated with TTR aggregates showed decreased metabolic activities as compared to TTR non-treated cells (Fong and Vieira, 2013). This indicates that the RNS-induced pro-oxidative effects could also hamper the metabolic activity of the cells. In a previous study by Saito et al. (2005), it was established that Wt and V30M (amyloidogenic variant) undergoes S-nitrosylation and due to which the proteins become amyloidogenic (Saito et al., 2005). Nitric oxide is generated in vessels from endothelial cells and smooth muscle cells. Vessels are the primary site for deposition of protein oligomers, therefore the nitrosylation of the TTR oligomers further enhances the ability to form amyloid fibrils which further contributes to the increased oxidative damage and *vice versa*. Since, there is a close correlation between TTR level and RNS or ROS, determination of TTR oligomeric level may help to evaluate the extent/magnitude of oxidative stress.

### TTR Oligomers Can Be Used to Detect Endoplasmic Reticulum (ER) Stress

When there is increased generation of misfolded or unfolded proteins in the ER, UPR initiates (Teixeira et al., 2006; Walter and Ron, 2011). This UPR is mediated by three signal pathways which involves inositol-requiring enzyme 1, activating transcription factor 6 and double-stranded RNA-activated protein kinase-like ER kinase (Walter and Ron, 2011; Minakshi et al., 2017; Rahman et al., 2017, 2018). Since TTR is an extracellular protein and it is unlikely that it may be involved in UPR generation in the ER. However, when TTR oligomers are deposited in neuronal cells, cardiomyocytes and kidney cells, there are chances that the misfolded species or TTR oligomers reach ER and cause oxidative stress. In a systemic study by Teixeira et al. (2006) the role of TTR oligomers in ER stress response was investigated using TTR transgenic mouse, and cell lines models (Teixeira et al., 2006). It was established that there was increased levels of BiP-immunoglobulin binding protein (an ER-resident chaperone) with extracellular TTR amyloid deposits in the brain of transgenic mouse. Furthermore using mouse neuronal ND7 cell line, it was also observed that oligomer-induced increased expression of BiP was mediated by the release of calcium from ER to cytosol (Teixeira et al., 2006). Because the activity of Caspases-3 was observed in cells treated with only TTR oligomers and not in cells treated with dantrolene and xestospongin (inhibitors of Ca^2+^ release). The involvement of other secondary messengers like D-myo inositol 1,4,5-triphosphate which receives signals at plasma membrane suggests that extracellular TTR oligomers have the potential to trigger ER stress in the surrounding cells. Taken together, the results convincingly supported the premise that the deposited TTR oligomers in tissues helps to induce UPR in the ER (Sekijima et al., 2005). Since TTR is a serum protein and is not present in ER, the study links the possibility of invoking UPR in ER by other extracellular proteins.

### Level of TTR Aggregates Negatively Correlates With the Activity of Catalase

Amyloid deposits consist of proteinaceous fibers which keep on depositing in tissues and form plaques. In case of AD, it was observed that these amyloid deposits had a toxic effect on cells as the cells showed apoptosis both *in vitro* and *in vivo*. Andersson et al. (2002) performed an *in vitro* study with different cell lines like neuroblastoma cell lines, PC12 cells, HeLa cells and some haematopoietic cell lines to investigate for the toxic effect of aggregated mutant TTR (Andersson et al., 2002). It was observed that the toxic effect was not cell specific and further it triggered the signaling cascade which ultimately led to apoptosis (Macedo et al., 2008). Interestingly, it has been shown that TTR induced apoptosis was inhibited by catalase in a concentration-dependent manner. Since catalase is an enzyme responsible for catalyzing H_2_O_2_ which is a predominant ROS, the results suggest that TTR oligomer-induced apoptosis is via production of H_2_O_2_ or other ROS.

### Oligomers of Different TTR Variants Exhibit Different Magnitude of Oxidative Stress

Protein oligomerization or amyloidogenesis has been considered to be one common hallmark of oxidative stress (Dobson, 1999). In fact, these protein oligomers are really toxic to the cells and can affect the integrity and hence function of various cell organelles (Zampagni et al., 2011). It is also believed that protein oligomers elevate the production of ROS which causes the oxidative stress and *vice versa* causing cell damage (Zempel et al., 2010). Protein oligomers can also force the release and normal function of Cytochrome C by directly affecting mitochondrial potential (Caroppi et al., 2009) or by affecting other pro-apoptotic molecules (Ott et al., 2007). Despite these developments, how different protein oligomers (or generated by different variants) could associate with the magnitude of proteotoxicity has not been explored yet. In this connection, TTR oligomers represents an emblemic signature as oligomers generated by different variants, is related to time of onset of disease pathology and hence determines the nature of proteotoxicity or oxidative stress (Quintas et al., 2001; Taguchi et al., 2013). For instance, Wt TTR oligomerization that leads to systemic senile amyloidosis was apparent in older individuals at the age of around 60–70 (Zhao et al., 2013). On the other hand, the pathogenic symptoms due to oligomers of the mutant variant V122I appeared early in age and patients die generally 10 years before the onset of senile systemic cardiomyopathy caused by Wt aggregation. The onset of familial amyloid polyneuropathy caused by the variant, V30M is around 25–33 years and death occurs 10 years after the onset (Koike et al., 2012; Takahashi et al., 2014; Arvidsson et al., 2015). L55P is considered to be most pathogenic variant of TTR and starts oligomerization at the physiological pH *in vitro* (as compared to the other mutants) and patients die at a very young age (Lashuel et al., 1999; Hammarström et al., 2002). Not only mutant variants, but Wt TTR has also been reported to undergo oxidation and carbonylation whose proteotoxicities (and hence oxidative stress) matches with the age of individuals. Thus, identification of different variants of TTR may be employed as a biomarker for the age related oxidative stress. Future research should focus on identification of newer TTR variants and their related onset of diseases or magnitude of oxidative stress.

## Summary and Future Perspectives

It is clearly evident from this review that different activity or post-translational modification of TTR is linked to specific disease pathologies *via* oxidative stress. The potential of TTR to cause oxidative stress is not only confined to serum, but also in ER. Therefore, in-depth insights to the various mechanism of oxidative stress induced by TTR and its oligomers will eventually lead to appropriate therapeutic strategies for these specific diseases. It is also understood that TTR oligomers can invoke different signaling cascades leading to different biological consequences (e.g., apoptosis, ROS and RNS generation, UPR and redox mediated oxidation) resulting in oxidative stress. It would therefore be important to explore the signaling cascade in detail by which oligomers help to induce such multiple consequences. Nevertheless TTR would be a potential biomarker of several human diseases linked with oxidative stress.

## Author Contributions

LS conceived the idea. MS, SK, and SR contributed to writing of the manuscript.

## Conflict of Interest Statement

The authors declare that the research was conducted in the absence of any commercial or financial relationships that could be construed as a potential conflict of interest.

## References

[B1] Abramov A. Y. Canevari L. Duchen M. R. (2004). Beta-amyloid peptides induce mitochondrial dysfunction and oxidative stress in astrocytes and death of neurons through activation of NADPH oxidase. *J. Neurosci.* 24 565–575. 10.1523/JNEUROSCI.4042-03.2004 14724257 PMC6729998

[B2] Andersson K. Olofsson A. Nielsen E. H. Svehag S. E. Lundgren E. (2002). Only amyloidogenic intermediates of transthyretin induce apoptosis. *Biochem. Biophs. Res. Commun.* 294 309–314. 10.1016/S0006-291X(02)00465-5 12051711

[B3] Arvidsson S. Pilebro B. Westermark P. Lindqvist P. Suhr O. B. (2015). Amyloid cardiomyopathy in hereditary transthyretin V30M amyloidosis - impact of sex and amyloid fibril composition. *PLoS One* 10:e0143456. 10.1371/journal.pone.0143456 26600306 PMC4658178

[B4] Basu T. K. Tze W. J. Leichter J. (1989). Serum vitamin A and retinol-binding protein in patients with insulin-dependent diabetes mellitus. *Am. J. Clin. Nutr.* 50 329–331. 10.1093/ajcn/50.2.329 2756919

[B5] Benson M. D. Kincaid J. C. (2007). The molecular biology and clinical features of amyloid neuropathy. *Muscle Nerve* 36 411–423. 10.1002/mus.20821 17554795

[B6] Brouillette J. Quirion R. (2008). Transthyretin: a key gene involved in the maintenance of memory capacities during aging. *Neurobiol. Aging* 29 1721–1732. 10.1016/j.neurobiolaging.2007.04.007 17512093

[B7] Butterfield D. A. Drake J. Pocernich C. Castegna A. (2001). Evidence of oxidative damage in Alzheimer’s disease brain: central role for amyloid beta-peptide. *Trends Mol. Med.* 7 548–554. 10.1016/S1471-4914(01)02173-6 11733217

[B8] Buxbaum J. N. Ye Z. Reixach N. Friske L. Levy C. Das P. (2008). Transthyretin protects Alzheimer’s mice from the behavioral and biochemical effects of abeta toxicity. *Proc. Natl. Acad. Sci. U.S.A.* 105 2681–2686. 10.1073/pnas.0712197105 18272491 PMC2268196

[B9] Caroppi P. Sinibaldi F. Fiorucci L. Santucci R. (2009). Apoptosis and human diseases: mitochondrion damage and lethal role of released cytochrome C as proapoptotic protein. *Curr. Med. Chem.* 16 4058–4065. 10.2174/092986709789378206 19754424

[B10] Cavallaro T. Martone R. L. Dwork A. J. Schon E. A. Herbert J. (1990). The retinal pigment epithelium is the unique site of transthyretin synthesis in the rat eye. *Invest. Ophthalmol. Vis. Sci.* 31 497–501. 1690688

[B11] Chen J. J. Genereux J. C. Suh E. H. Vartabedian V. F. Rius B. Qu S. (2016). Endoplasmic reticulum proteostasis influences the oligomeric state of an amyloidogenic protein secreted from mammalian cells. *Cell Chem. Biol.* 23 1282–1293. 10.1016/j.chembiol.2016.09.001 27720586 PMC5108364

[B12] Colon W. Kelly J. W. (1992). Partial denaturation of transthyretin is sufficient for amyloid fibril formation in vitro. *Biochemistry* 31 8654–8660. 10.1021/bi00151a036 1390650

[B13] Costa P. P. Figueira A. S. Bravo F. R. (1978). Amyloid fibril protein related to prealbumin in familial amyloidotic polyneuropathy. *Proc. Natl. Acad. Sci. U.S.A.* 75 4499–4503. 10.1073/pnas.75.9.4499 279930 PMC336143

[B14] Costa R. Ferreira-Da-Silva F. Saraiva M. J. Cardoso I. (2008). Transthyretin protects against A-beta peptide toxicity by proteolytic cleavage of the peptide: a mechanism sensitive to the kunitz protease inhibitor. *PLoS One* 3:e2899. 10.1371/journal.pone.0002899 18682830 PMC2483353

[B15] Dalle-Donne I. Rossi R. Colombo G. Giustarini D. Milzani A. (2009). Protein S-glutathionylation: a regulatory device from bacteria to humans. *Trends Biochem. Sci.* 34 85–96. 10.1016/j.tibs.2008.11.002 19135374

[B16] de Sousa M. M. Vital C. Ostler D. Fernandes R. Pouget-Abadie J. Carles D. (2000). Apolipoprotein AI and transthyretin as components of amyloid fibrils in a kindred with apoAI Leu178His amyloidosis. *Am. J. Pathol.* 156 1911–1917. 10.1016/S0002-9440(10)65064-X 10854214 PMC1850097

[B17] Dobson C. M. (1999). Protein misfolding, evolution and disease. *Trends Biochem. Sci.* 24 329–332. 10.1016/S0968-0004(99)01445-0 10470028

[B18] Escher N. Kaatz M. Melle C. Hipler C. Ziemer M. Driesch D. (2007). Posttranslational modifications of transthyretin are serum markers in patients with mycosis fungoides. *Neoplasia* 9 254–259. 10.1593/neo.06805 17401465 PMC1838582

[B19] Fleming C. E. Saraiva M. J. Sousa M. M. (2007). Transthyretin enhances nerve regeneration. *J. Neurochem.* 103 831–839. 10.1111/j.1471-4159.2007.04828.x 17897357

[B20] Fong V. H. Vieira A. (2013). Transthyretin aggregates induce production of reactive nitrogen species. *Neurodegener Dis.* 11 42–48. 10.1159/000338153 22627469

[B21] Genereux J. C. Qu S. Zhou M. Ryno L. M. Wang S. Shoulders M. D. (2015). Unfolded protein response-induced ERdj3 secretion links ER stress to extracellular proteostasis. *EMBO J.* 34 4–19. 10.15252/embj.201488896 25361606 PMC4291477

[B22] Ghezzi P. (2005). Oxidoreduction of protein thiols in redox regulation. *Biochem. Soc. Trans.* 33 1378–1381. 10.1042/BST0331378 16246123

[B23] Gimeno A. Santos L. M. Alemi M. Rivas J. Blasi D. Cotrina E. Y. (2017). Insights on the interaction between transthyretin and abeta in solution. a saturation transfer difference (STD) nmr analysis of the role of iododiflunisal. *J. Med. Chem.* 60 5749–5758. 10.1021/acs.jmedchem.7b00428 28587455

[B24] Gonzalez G. Offord R. E. (1971). The subunit structure of prealbumin. *Biochem. J.* 125 309–317. 10.1042/bj1250309 4110459 PMC1178055

[B25] Gordon D. J. Probstfield J. L. Garrison R. J. Neaton J. D. Castelli W. P. Knoke J. D. (1989). High-density lipoprotein cholesterol and cardiovascular disease. Four prospective American studies. *Circulation* 79 8–15. 10.1161/01.CIR.79.1.8 2642759

[B26] Hammarström P. Jiang X. Hurshman A. R. Powers E. T. Kelly J. W. (2002). Sequence-dependent denaturation energetics: a major determinant in amyloid disease diversity. *Proc. Natl. Acad. Sci. U.S.A.* 99(Suppl. 4), 16427–16432. 10.1073/pnas.202495199 12351683 PMC139904

[B27] Ingbar S. H. (1958). Pre-albumin: a thyroxinebinding protein of human plasma. *Endocrinology* 63 256–259. 10.1210/endo-63-2-256 13562035

[B28] Jacobson D. R. Pastore R. D. Yaghoubian R. Kane I. Gallo G. Buck F. S. (1997). Variant-sequence transthyretin (isoleucine 122) in late-onset cardiac amyloidosis in black Americans. *N. Engl. J. Med.* 336 466–473. 10.1056/NEJM199702133360703 9017939

[B29] Kanda Y. Goodman D. S. Canfield R. E. Morgan F. J. (1974). The amino acid sequence of human plasma prealbumin. *J. Biol. Chem.* 249 6796–6805. 4607556

[B30] Kaplowitz N. (1981). The importance and regulation of hepatic glutathione. *Yale J. Biol. Med.* 54 497–502. 7342494 PMC2596047

[B31] Kawaji T. Ando Y. Nakamura M. Yamamoto K. Ando E. Takano A. (2005). Transthyretin synthesis in rabbit ciliary pigment epithelium. *Exp. Eye Res.* 81 306–312. 10.1016/j.exer.2005.02.003 16129098

[B32] Koike H. Tanaka F. Hashimoto R. Tomita M. Kawagashira Y. Iijima M. (2012). Natural history of transthyretin Val30Met familial amyloid polyneuropathy: analysis of late-onset cases from non-endemic areas. *J. Neurol. Neurosurg. Psychiatry* 83 152–158. 10.1136/jnnp-2011-301299 22228785

[B33] Lashuel H. A. Wurth C. Woo L. Kelly J. W. (1999). The most pathogenic transthyretin variant, L55P, forms amyloid fibrils under acidic conditions and protofilaments under physiological conditions. *Biochemistry* 38 13560–13573. 10.1021/bi991021c 10521263

[B34] Li X. Masliah E. Reixach N. Buxbaum J. N. (2011). Neuronal production of transthyretin in human and murine Alzheimer’s disease: is it protective? *J. Neurosci.* 31 12483–12490. 10.1523/JNEUROSCI.2417-11.2011 21880910 PMC3172869

[B35] Lightfoot T. J. Skibola C. F. Smith A. G. Forrest M. S. Adamson P. J. Morgan G. J. (2006). Polymorphisms in the oxidative stress genes, superoxide dismutase, glutathione peroxidase and catalase and risk of non-Hodgkin’s lymphoma. *Haematologica* 91 1222–1227. 16956821

[B36] Liz M. A. Faro C. J. Saraiva M. J. Sousa M. M. (2004). Transthyretin, a new cryptic protease. *J. Biol. Chem.* 279 21431–21438. 10.1074/jbc.M402212200 15033978

[B37] Liz M. A. Gomes C. M. Saraiva M. J. Sousa M. M. (2007). ApoA-I cleaved by transthyretin has reduced ability to promote cholesterol efflux and increased amyloidogenicity. *J. Lipid Res.* 48 2385–2395. 10.1194/jlr.M700158-JLR200 17693625

[B38] Macedo B. Batista A. R. Ferreira N. Almeida M. R. Saraiva M. J. (2008). Anti-apoptotic treatment reduces transthyretin deposition in a transgenic mouse model of familial amyloidotic polyneuropathy. *Biochim. Biophys. Acta* 1782 517–522. 10.1016/j.bbadis.2008.05.005 18572024

[B39] Marques C. A. Keil U. Bonert A. Steiner B. Haass C. Muller W. E. (2003). Neurotoxic mechanisms caused by the Alzheimer’s disease-linked swedish amyloid precursor protein mutation: oxidative stress, caspases, and the JNK pathway. *J. Biol. Chem.* 278 28294–28302. 10.1074/jbc.M212265200 12730216

[B40] Martinho A. Goncalves I. Costa M. Santos C. R. (2012). Stress and glucocorticoids increase transthyretin expression in rat choroid plexus via mineralocorticoid and glucocorticoid receptors. *J. Mol. Neurosci.* 48 1–13. 10.1007/s12031-012-9715-7 22371232

[B41] Minakshi R. Rahman S. Jan A. T. Archana A. Kim J. (2017). Implications of aging and the endoplasmic reticulum unfolded protein response on the molecular modality of breast cancer. *Exp. Mol. Med.* 49:e389. 10.1038/emm.2017.215 29123254 PMC5704197

[B42] Navab M. Hama S. Y. Anantharamaiah G. M. Hassan K. Hough G. P. Watson A. D. (2000). Normal high density lipoprotein inhibits three steps in the formation of mildly oxidized low density lipoprotein: steps 2 and 3. *J. Lipid Res.* 41 1495–1508. 10974057

[B43] Naylor H. M. Newcomer M. E. (1999). The structure of human retinol-binding protein (RBP) with its carrier protein transthyretin reveals an interaction with the carboxy terminus of RBP. *Biochemistry* 38 2647–2653. 10.1021/bi982291i 10052934

[B44] Nilsson L. Pamren A. Islam T. Brannstrom K. Golchin S. A. Pettersson N. (2018). Transthyretin interferes with abeta amyloid formation by redirecting oligomeric nuclei into non-amyloid aggregates. *J. Mol. Biol.* 430 2722–2733. 10.1016/j.jmb.2018.06.005 29890120

[B45] Ott M. Gogvadze V. Orrenius S. Zhivotovsky B. (2007). Mitochondria, oxidative stress and cell death. *Apoptosis* 12 913–922. 10.1007/s10495-007-0756-2 17453160

[B46] Podrez E. A. (2010). Anti-oxidant properties of high-density lipoprotein and atherosclerosis. *Clin. Exp. Pharmacol. Physiol.* 37 719–725. 10.1111/j.1440-1681.2010.05380.x 20374263 PMC3010184

[B47] Power D. M. Elias N. P. Richardson S. J. Mendes J. Soares C. M. Santos C. R. (2000). Evolution of the thyroid hormone-binding protein, transthyretin. *Gen. Comp. Endocrinol.* 119 241–255. 10.1006/gcen.2000.7520 11017772

[B48] Quintas A. Vaz D. C. Cardoso I. Saraiva M. J. Brito R. M. (2001). Tetramer dissociation and monomer partial unfolding precedes protofibril formation in amyloidogenic transthyretin variants. *J. Biol. Chem.* 276 27207–27213. 10.1074/jbc.M101024200 11306576

[B49] Rahman S. Archana A. Jan A. T. Minakshi R. (2018). Dissecting endoplasmic reticulum unfolded protein response (UPR(ER)) in managing clandestine modus operandi of alzheimer’s disease. *Front. Aging Neurosci.* 10:30. 10.3389/fnagi.2018.00030 29467648 PMC5808164

[B50] Rahman S. Jan A. T. Ayyagari A. Kim J. Kim J. Minakshi R. (2017). Entanglement of UPRER in aging driven neurodegenerative diseases. *Front. Aging Neurosci.* 9:341. 10.3389/fnagi.2017.00341 29114219 PMC5660724

[B51] Raz A. Goodman D. S. (1969). The interaction of thyroxine with human plasma prealbumin and with the prealbumin-retinol-binding protein complex. *J. Biol. Chem.* 244 3230–3237. 4978316

[B52] Ribeiro C. A. Saraiva M. J. Cardoso I. (2012). Stability of the transthyretin molecule as a key factor in the interaction with a-beta peptide–relevance in Alzheimer’s disease. *PLoS One* 7:e45368. 10.1371/journal.pone.0045368 23028965 PMC3444465

[B53] Saito S. Ando Y. Nakamura M. Ueda M. Kim J. Ishima Y. (2005). Effect of nitric oxide in amyloid fibril formation on transthyretin-related amyloidosis. *Biochemistry* 44 11122–11129. 10.1021/bi050327i 16101296

[B54] Sant’anna R. Almeida M. R. Varejao N. Gallego P. Esperante S. Ferreira P. (2017). Cavity filling mutations at the thyroxine-binding site dramatically increase transthyretin stability and prevent its aggregation. *Sci. Rep.* 7:44709. 10.1038/srep44709 28338000 PMC5364509

[B55] Saraiva M. J. Magalhaes J. Ferreira N. Almeida M. R. (2012). Transthyretin deposition in familial amyloidotic polyneuropathy. *Curr. Med. Chem.* 19 2304–2311. 10.2174/092986712800269236 22471982

[B56] Sasaki H. Yoshioka N. Takagi Y. Sakaki Y. (1985). Structure of the chromosomal gene for human serum prealbumin. *Gene* 37 191–197. 10.1016/0378-1119(85)90272-0 4054629

[B57] Schwarzman A. L. Gregori L. Vitek M. P. Lyubski S. Strittmatter W. J. Enghilde J. J. (1994). Transthyretin sequesters amyloid beta protein and prevents amyloid formation. *Proc. Natl. Acad. Sci. U.S.A.* 91 8368–8372. 10.1073/pnas.91.18.8368 8078889 PMC44607

[B58] Sekijima Y. Wiseman R. L. Matteson J. Hammarstrom P. Miller S. R. Sawkar A. R. (2005). The biological and chemical basis for tissue-selective amyloid disease. *Cell* 121 73–85. 10.1016/j.cell.2005.01.018 15820680

[B59] Silva C. S. Eira J. Ribeiro C. A. Oliveira A. Sousa M. M. Cardoso I. (2017). Transthyretin neuroprotection in Alzheimer’s disease is dependent on proteolysis. *Neurobiol. Aging* 59 10–14. 10.1016/j.neurobiolaging.2017.07.002 28780366

[B60] Soprano D. R. Herbert J. Soprano K. J. Schon E. A. Goodman D. S. (1985). Demonstration of transthyretin mRNA in the brain and other extrahepatic tissues in the rat. *J. Biol. Chem.* 260 11793–11798. 4044580

[B61] Sousa M. M. Berglund L. Saraiva M. J. (2000). Transthyretin in high density lipoproteins: association with apolipoprotein A-I. *J. Lipid. Res.* 41 58–65. 10627502

[B62] Sousa M. M. Cardoso I. Fernandes R. Guimaraes A. Saraiva M. J. (2001). Deposition of transthyretin in early stages of familial amyloidotic polyneuropathy: evidence for toxicity of nonfibrillar aggregates. *Am. J. Pathol.* 159 1993–2000. 10.1016/S0002-9440(10)63050-7 11733349 PMC1850610

[B63] Sparkes R. S. Sasaki H. Mohandas T. Yoshioka K. Klisak I. Sakaki Y. (1987). Assignment of the prealbumin (PALB) gene (familial amyloidotic polyneuropathy) to human chromosome region 18q11.2-q12.1. *Hum. Genet.* 75 151–154. 10.1007/BF00591077 3028932

[B64] Taguchi K. Jono H. Kugimiya-Taguchi T. Nagao S. Su Y. Yamasaki K. (2013). Effect of albumin on transthyretin and amyloidogenic transthyretin Val30Met disposition and tissue deposition in familial amyloidotic polyneuropathy. *Life Sci.* 93 1017–1022. 10.1016/j.lfs.2013.10.031 24211615

[B65] Takahashi R. Ono K. Shibata S. Nakamura K. Komatsu J. Ikeda Y. (2014). Efficacy of diflunisal on autonomic dysfunction of late-onset familial amyloid polyneuropathy (TTR Val30Met) in a Japanese endemic area. *J. Neurol. Sci.* 345 231–235. 10.1016/j.jns.2014.07.017 25060417

[B66] Teixeira P. F. Cerca F. Santos S. D. Saraiva M. J. (2006). Endoplasmic reticulum stress associated with extracellular aggregates. Evidence from transthyretin deposition in familial amyloid polyneuropathy. *J. Biol. Chem.* 281 21998–22003. 10.1074/jbc.M602302200 16751191

[B67] Terazaki H. Ando Y. Suhr O. Ohlsson P. I. Obayashi K. Yamashita T. (1998). Post-translational modification of transthyretin in plasma. *Biochem. Biophys. Res. Commun.* 249 26–30. 10.1006/bbrc.1998.9097 9705825

[B68] Toborek M. Hennig B. (1994). Fatty acid-mediated effects on the glutathione redox cycle in cultured endothelial cells. *Am. J. Clin. Nutr.* 59 60–65. 10.1093/ajcn/59.1.60 8279404

[B79] Ton V. K. Mukherjee M. Judge D. P. (2014). Transthyretin cardiac amyloidosis: pathogenesis, treatments, and emerging role in heart failure with preserved ejection fraction. *Clin. Med. Insights Cardiol.* 8 39’44. 10.4137/CMC.S15719 25628512 PMC4284988

[B69] Tsuzuki T. Mita S. Maeda S. Araki S. Shimada K. (1985). Structure of the human prealbumin gene. *J. Biol. Chem.* 260 12224–12227. 2995367

[B70] Wakasugi S. Maeda S. Shimada K. (1986). Structure and expression of the mouse prealbumin gene. *J. Biochem.* 100 49–58. 10.1093/oxfordjournals.jbchem.a121705 3020014

[B71] Walter P. Ron D. (2011). The unfolded protein response: from stress pathway to homeostatic regulation. *Science* 334 1081–1086. 10.1126/science.1209038 22116877

[B72] Westermark G. T. Westermark P. (2008). Transthyretin and amyloid in the islets of Langerhans in type-2 diabetes. *Exp. Diabetes Res.* 2008:429274. 10.1155/2008/429274 18825272 PMC2553203

[B73] Westermark P. Sletten K. Johansson B. Cornwell G. G. III (1990). Fibril in senile systemic amyloidosis is derived from normal transthyretin. *Proc. Natl. Acad. Sci. U.S.A.* 87 2843–2845. 10.1073/pnas.87.7.2843 2320592 PMC53787

[B74] Yang Q. Graham T. E. Mody N. Preitner F. Peroni O. D. Zabolotny J. M. (2005). Serum retinol binding protein 4 contributes to insulin resistance in obesity and type 2 diabetes. *Nature* 436 356–362. 10.1038/nature03711 16034410

[B75] Yokoyama T. Takaki S. Chosa K. Sato T. Suico M. A. Teranishi Y. (2015). Structural stabilization of transthyretin by a new compound, 6-benzoyl-2-hydroxy-1H-benzo[de]isoquinoline-1,3(2H)-dione. *J. Pharmacol. Sci.* 129 240–243. 10.1016/j.jphs.2015.09.006 26639444

[B76] Zampagni M. Cascella R. Casamenti F. Grossi C. Evangelisti E. Wright D. (2011). A comparison of the biochemical modifications caused by toxic and non-toxic protein oligomers in cells. *J. Cell. Mol. Med.* 15 2106–2116. 10.1111/j.1582-4934.2010.01239.x 21155974 PMC4394221

[B77] Zempel H. Thies E. Mandelkow E. Mandelkow E. M. (2010). Abeta oligomers cause localized Ca(2+) elevation, missorting of endogenous tau into dendrites, tau phosphorylation, and destruction of microtubules and spines. *J. Neurosci.* 30 11938–11950. 10.1523/JNEUROSCI.2357-10.2010 20826658 PMC6633549

[B78] Zhao L. Buxbaum J. N. Reixach N. (2013). Age-related oxidative modifications of transthyretin modulate its amyloidogenicity. *Biochemistry* 52 1913–1926. 10.1021/bi301313b 23414091 PMC3604100

